# Pilomatrixoma in the head and neck

**DOI:** 10.1016/S1808-8694(15)30504-8

**Published:** 2015-10-19

**Authors:** José Arruda Mendes Neto, Rafael Mônaco Raposo, Danilo Kanashiro Segalla, Fernando Danelon Leonhardt

**Affiliations:** 1 ENT Resident Physician - Federal University of São Paulo - Paulista School of Medicine; 2 Otorhinolaryngologist. Rhinology fellow - Department of Otorhinolaryngology - Head and Neck Surgery - Federal University of São Paulo - Paulista School of Medicine; 3 Resident Physician; 4 MSc. PhD Student - Head and Neck program - Department of Otorhinolaryngology - Head and Neck Surgery - Federal University of São Paulo - Paulista School of Medicine. Federal University of São Paulo - Paulista School of Medicine

**Keywords:** head and neck neoplasms, pilomatrixoma

## INTRODUCTION

Malherbe and Chenantais were the first to report a pilomatrixoma in 1880. They described a calcifying epithelioma, believing it to be a sebaceous gland tumor. Since 1905, this uncommon neoplasia has been called Malherbe's calcifying epithelioma. Numerous studies suggest that this tumor stems from the external sheath of hair follicles. In 1961, Forbis and Helwig proposed the term pilomatrixoma, which is more etymologically correct^1^. It is a benign skin neoplasia, originating from hair follicle matrix cells^2^, representing 0.12% of skin tumors^3^. In this study we present two cases of pilomatrixoma and discuss the main aspects of this neoplasia.

## CASE PRESENTATION

### Case 1

**Figure 1:**
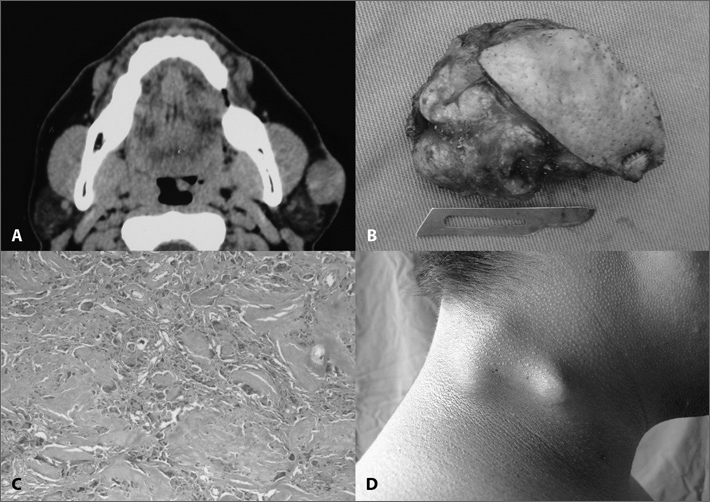
A: CT scan axial view of a face showing a nodular growth of about 4cm in its longest axis, subcutaneously located just above the left parotid gland, in close contact with it, highlighted by the intravenous contrast. B: Macroscopic view of the lesion after resection. C: histologic aspect of the pilomatrixoma. D. Tumor appearance of patient #2, before resection.

A 39 year old male patient, with a painless, slow growth left pre-auricular nodule evolving for one year. On physical exam we noticed a tumor in the left pre-auricular region, measuring 2.5 × 1.5cm, hard, somewhat fix. A face CT scan showed a 4cm nodular tumor located in the subcutaneous tissue above the parotid gland, in close contact with it, highlighted by endovenous contrast (Fig. 1A). A fine needle aspiration (FNA) showed thick plates of necrotic squamous cells amidst neutrophils, lymphocytes and multinucleated giant cells with fibrosis, suggesting pilomatrixoma. After FNA review, we considered the possibility of a well differentiated squamous cell carcinoma metastasis. The nodule was resected together with superficial parotidectomy because of the possibility of a carcinoma (Figure 1B). During surgery, the specimen was sent to histology, which confirmed pilomatrixoma (Figure 1C). With one year of follow up, there were no signs of recurrence.

### Case 2

An 18 year old male had had a painless and slow growth neck nodule for two years. On physical exam, he had a level V tumor on the right side, of 2 × 1cm in size, hard, somewhat fixed and painless (Figure 1D). FNA showed a polymorphic population of basaloid cells of squamous aspect, gigantic multinucleated cells and ghost cells, thus suggesting pilomatrixoma. The lesion was resected and there were no relapses after two years of follow up.

## DISCUSSION

The pilomatrixoma is a relatively rare skin neoplasia. It may affect individuals at any age, with incidence peaks on the first and sixth decades of life, and it is more common in women (1.5 to 2.5:1). Among young people, 40% happen before 10 years of age and 60% before 201,4. In the cases hereby presented, the patients had 39 and 18 years of age and were males.

New hair follicles are not formed after birth, only some are activated during puberty. If they are located in very deep layers, differentiation induction agents will not act properly on them. These partially differentiated follicles would form the pilomatrixomas^1^.

The most frequent location is on the neck, followed by the frontal, periorbital and pre-auricular regions. Clinically it manifests as a subcutaneous or intradermal, hard and slow growth tumor^3^.

The pre-surgical diagnosis is almost always difficult, especially when the elderly are concerned. The finding of a keratinous material in the FNA can be misinterpreted as carcinoma^2^, ^3^, ^5^, ^6^.

Histological characteristics include ghost cells in the center with basophilic nucleated cells in the periphery. Calcification is present in 70-95%^1^, ^3^. The presence of nuclear pleomorphism, atypical mitosis, central necrosis, skin and adjacent tissue infiltration, besides ulceration, is suggestive of malignancy^6^.

The CT scan has little value to approach pilomatrixomas. It is mainly used to differentiate pre-auricular from parotid tumors and to assess large and aggressive tumors. The characteristic is a well outlined and calcified subcutaneous lesion^3^, similar to the one found in our cases.

Standard treatment involves tumor resection. It is recommended to remove the tumor with safety margins in order to minimize the risk of recurrence of the malignant variants. Adjacent skin must be occasionally resected when adhered to the dermis. Recurrences are rare, occurring in 0 a 3%^1^, ^2^, ^3^, ^4^, ^5^, ^6^.

## FINAL REMARKS

Although being a benign neoplasia, pilomatrixomas can be misdiagnosed as carcinoma. It is important that the otorhinolaryngologist be familiarized with this neoplasia, and considers it in the differential diagnosis of a superficial tumor in the head and neck region, thus avoiding unnecessary aggressive resections.

## References

[1] Fernandes R, Holmes J, Mullenix C (2003). Giant pilomatricoma (epithelioma of Melherbe): report of a case and review of literature. J Oral Maxillofac Surg..

[2] Greene RM, McGuff HS, Miller FR (2004). Pilomatrixoma of the face:a benign skin appendage mimicking squamous cell carcinoma. Otolaryngol Head Neck Surg..

[3] Lan MY, Lan MC, Ho CY, Li WY, Lin CZ (2003). Pilomatricoma of the head and neck:a retrospective review of 179 cases. Arch Otolaryngol Head Neck Surg..

[4] Pirouzmanesh A, Reinisch JF, Gonzalez-Gomez I, Smith EM, Meara JG (2003). Pilomatrixoma:a review of 346 cases. Plast Reconstr Surg..

[5] Saussez S, Mahillon V, Blaivie C, Haller A, Chantrain G, Thill MP (2005). Aggressive pilomatrixoma of the infra-auricular area:a case report. Auris Nasus Larynx..

[6] Phyu KK, Bradley PJ (2001). Pilomatrixoma in the parotid region. J Laryngol Otol..

